# Targeting the ubiquitination/deubiquitination process to regulate immune checkpoint pathways

**DOI:** 10.1038/s41392-020-00418-x

**Published:** 2021-01-22

**Authors:** Jiaxin Liu, Yicheng Cheng, Ming Zheng, Bingxiao Yuan, Zimu Wang, Xinying Li, Jie Yin, Mingxiang Ye, Yong Song

**Affiliations:** 1 Department of Respiratory and Critical Care Medicine, Affiliated Jinling Hospital, Medical School of Nanjing University, 210002 Nanjing, Jiangsu China; 2 Department of Respiratory and Critical Care Medicine, Jinling Hospital, Nanjing University School of Medicine, 210002 Nanjing, China; 3 Department of Stomatology, Jinling Hospital, Medical School of Nanjing University, Nanjing, 210002 China; 4 Department of Respiratory and Critical Care Medicine, Jinling Hospital, Nanjing Medical University, 210002 Nanjing, Jiangsu China

**Keywords:** Immunotherapy, Cancer, Immunological disorders

## Abstract

The immune system initiates robust immune responses to defend against invading pathogens or tumor cells and protect the body from damage, thus acting as a fortress of the body. However, excessive responses cause detrimental effects, such as inflammation and autoimmune diseases. To balance the immune responses and maintain immune homeostasis, there are immune checkpoints to terminate overwhelmed immune responses. Pathogens and tumor cells can also exploit immune checkpoint pathways to suppress immune responses, thus escaping immune surveillance. As a consequence, therapeutic antibodies that target immune checkpoints have made great breakthroughs, in particular for cancer treatment. While the overall efficacy of immune checkpoint blockade (ICB) is unsatisfactory since only a small group of patients benefited from ICB treatment. Hence, there is a strong need to search for other targets that improve the efficacy of ICB. Ubiquitination is a highly conserved process which participates in numerous biological activities, including innate and adaptive immunity. A growing body of evidence emphasizes the importance of ubiquitination and its reverse process, deubiquitination, on the regulation of immune responses, providing the rational of simultaneous targeting of immune checkpoints and ubiquitination/deubiquitination pathways to enhance the therapeutic efficacy. Our review will summarize the latest findings of ubiquitination/deubiquitination pathways for anti-tumor immunity, and discuss therapeutic significance of targeting ubiquitination/deubiquitination pathways in the future of immunotherapy.

## Introduction

The immune system, including the innate and adaptive immune systems, helps to defend against pathogens and tumors and prevent damage to tissues, thus maintaining organismal homeostasis. Given that overactive immune responses can be detrimental (inflammatory or autoimmune diseases), immune checkpoints, which are a set of molecules including cytotoxic T lymphocyte-associated protein-4 (CTLA-4), programmed cell-death protein-1(PD-1), lymphocyte activation gene-3 (LAG-3), T cell immunoglobulin and ITIM domain (TIGIT), and T cell immunoglobulin and mucin domain-containing-3 (TIM-3), are needed to suppress excessive immune responses, maintain self-tolerance and avoid self-damage.^1^ However, tumors can also utilize the immune checkpoint pathways to inhibit anti-tumor immune response and evade immune surveillance, eventually resulting in tumor outgrowth and progression.^2^ Hence, drugs that target immune checkpoints have been developed over recent decades. In particular, the use of immune checkpoint blockade (ICB) has achieved great success in cancer therapy since the first ICB treatment, ipilimumab (anti-CTLA-4), was approved by the U.S. Food and Drug Administration (FDA) in 2011 for the treatment of metastatic melanoma.^3^ Nevertheless, ICB treatment is still limited due to low response rates, de novo or acquired resistance and inevitable immunotherapy-related adverse events (irAEs).^4–7^ Therefore, there is an urgent need to identify new strategies that can be combined with ICB treatments to improve their efficacy and overcome the challenges in the era of immunotherapy.

Ubiquitination is a highly conserved posttranslational modification in mammals. Ubiquitination is a stepwise process that is carried out by ubiquitin-activating enzyme E1, ubiquitin-conjugating enzyme E2, and ubiquitin ligase E3. Ubiquitination involves the transfer of the C-terminal glycine of ubiquitin to an ε-NH2 group of a lysine residue in a substrate.^8,9^ Ubiquitination can be classified into three major categories, including monoubiquitination, multiubiquitination, and polyubiquitination, which results in proteolysis and signal transduction. In contrast, ubiquitination can be reversed by deubiquitinases (DUBs) via the removal of ubiquitin chains, leading to the termination of ubiquitination and the preservation of substrate protein expression.^10,11^ The interaction between ubiquitination and deubiquitination plays crucial roles in almost all aspects of biological activities.^12^ Strikingly, multiple processes involved in innate and adaptive immunity, such as antigen presentation, cell differentiation, immune defense, and inflammatory responses, are regulated by ubiquitination/ deubiquitination.^13–15^

Taken together, the ubiquitination/deubiquitination pathways may become potential therapeutic targets for the treatment of cancers, infections, and autoimmune diseases. Our review will summarize the research progress and the latest findings about the interactions between immune checkpoint pathways and ubiquitination/deubiquitination in cancers, infections, and autoimmune diseases, and discuss targeting ubiquitination/deubiquitination as a potential strategy for immunotherapy.

## Immune checkpoints

The full activation of T cells requires the simultaneous coactivation of three signalings: (1) the T cell receptor (TCR) binds to a major histocompatibility complex (MHC)–peptide complex presented by antigen-presenting cells (APCs); (2) CD80/CD86 expressed by APCs binds to the costimulatory molecule CD28 expressed by T cells; and (3) cytokines either enhance or suppress immune responses.^16–18^ However, overactivation of the immune response leads to a series of human illnesses, including autoimmune diseases.^10^ Therefore, there are negative feedback mechanisms which function as brakes to limit the activity of T cells and suppress immune cell-mediated tissue damage.^19^ These brakes are known as immune checkpoints and are important mechanisms for maintaining homeostasis under physiological conditions; nevertheless, cancer cells may utilize these immune checkpoints to evade immune surveillance and promote tumor outgrowth (Table 1).^2^ Thus, the blockade of immune checkpoint molecules has become a novel treatment strategy that utilizes the host’s own immune system to kill cancer cells.^20^ Indeed, tremendous efforts have been made to develop immune checkpoint inhibitors (ICIs) and test their safety and efficacy in various human malignancies. Given the great success of anti-CTLA4 and anti-PD-1/PD-L1 therapies in a panel of multicenter randomized clinical trials, several ICIs have been approved by the FDA as front-line treatments for melanoma,^21–23^ non-small cell lung cancer (NSCLC)^24,25^ and other cancers.^4^

**Table 1:** A summary of immune checkpoint molecules

Molecules	Full name	Alternate name	Binding partners	Expressed cells	References
CTLA-4	Cytotoxic T lymphocyte-associated protein-4	CD152	CD80 (B7-1) CD86 (B7-2)	T cells	^5,139^
PD-1	Programmed cell-death protein-1	CD279	PD-L1 (B7-H1 or CD274) PD-L2 (B7-DC or CD273)	T cells, NKT cells, B cells, monocytes, Langerhans’ cells	^16,140^
LAG-3	Lymphocyte activation gene-3	CD223	MHC-II FGL1 galectin-3 (LGALS3) LSECtin α-synuclein	T cells, B cells, Tregs, NK cells, NKT cells, pDCs	^5,141,142^
TIGIT	T-cell immunoglobulin and ITIM domain	WUCAM Vstm3 Vsig9	CD155 (PVR, Necl-5) CD112 (nectin-2, PRR2, PVRL2)	T cells, Tregs, NK cells,	^143–145^
TIM-3	T-cell immunoglobulin and mucin-domain containing-3	HAVCR2 CD366	Galectin-9 Ceacam-1 HMGB-1 PtdSer	T cells, Tregs, DCs, NK cells, monocytes, macrophages	^5,18,144–146^
BTLA	B and T cell lymphocyte attenuator	CD272	HVEM	B cells, T cells, macrophages, DCs	^147,148^
VISTA	V-domain Ig suppressor of T cell activation	PD-1H DD1α Gi24 Dies1 B7-H5	VSIG-3	APCs, T cells, Tregs	^5,149^
B7-H3	B7 homolog 3 protein	CD276	IL20RA TLT2 (putative)	T cells, B cells, APCs, NK cells, DCs, monocytes, fibroblasts, epithelial cells, tumor cells	^5,145,150–153^
B7-H4	B7 homolog 4 protein	VTC1 B7x B7S1	unknown	T cells, B cells, monocytes, DCs	^145,154,155^
KIR	Killer-cell immunoglobulin-like receptor	CD158	MHC class I molecules	NK cells, T cells	^111,156^
CD160	Cluster of differentiation 160	–	HVEM	NK cells, T cells, intraepithelial lymphocytes	^157^
CD73	Cluster of differentiation 73	ecto-5’- nucleotidase	Dephosphorylating extracellular AMP to generate adenosine	Tumor cells, Th cells, Tregs, T cells, B cells, macrophages, DCs	^158,159^
CD96	Cluster of differentiation 96	–	CD111 CD155	T cells, NK cells, NKT cells	^160,161^

Unfortunately, only a small group of cancer patients can benefit from ICIs, and the overall response rate to ICI single-agent therapy is less than 30%. In addition, there are emerging challenges regarding resistance to ICIs and irAEs in clinical settings.^4^ Identification of biomarkers that predict the response to ICI therapy would help clinicians to select patients who would benefit from ICI therapy and elucidate the mechanisms of resistance. We and others have demonstrated that protein ubiquitination/deubiquitination not only results in proteolysis, but also mediates cell growth signaling transduction. Disruption of ubiquitination/deubiquitination has been implicated in many cancers and is associated with tumor metastasis and resistance to cytotoxic agents.^26–29^ Given the pivotal role of ubiquitination/deubiquitination in cancer cell proliferation and resistance to apoptosis, we predict that targeting ubiquitination/deubiquitination may be a feasible way to sensitize cells to immunotherapy and overcome resistance to ICIs.^30^

## Ubiquitination and deubiquitination

Protein ubiquitination is a stepwise biological event in which the ubiquitin molecule is tagged onto a lysine site of a substrate protein. Protein ubiquitination is initiated by ubiquitin-activating enzyme E1, and the cysteine residue of E1 binds to the C-terminal glycine residue of ubiquitin in an ATP-dependent manner. Next, ubiquitin-conjugating enzyme E2 catalyzes the transfer of activated ubiquitin from E1 to a cysteine residue of E2. In the final step, ubiquitin ligase E3 catalyzes the transfer of ubiquitin by forming an isopeptide bond between the lysine ε-amino group of the substrate and the C-terminal glycine of ubiquitin.^8,31^ It has been reported that the E1 family has only two members, and the E2 family has approximately 35 members, while there are over 600 E3 ligases. Notably, E3 determines the substrate specificity in the ubiquitination process.^10^

**Fig. 1:**
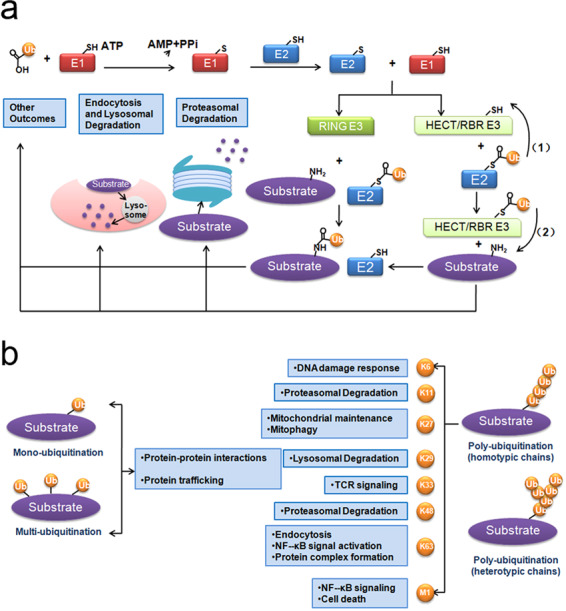
Schematic diagram of ubiquitination. **a** The biological events during ubiquitination. **b** The classification of ubiquitination and corresponding biological functions

E3s can be further divided into three categories based on the specific domains through which ubiquitin is transferred to the target proteins: (1) homologous to the E6-associated protein C-terminus (HECT) domain-containing E3s, which themselves accept ubiquitin onto a cysteine residue by forming a thiol ester bond before transferring ubiquitin to the substrates; (2) really interesting new gene (RING) domain-containing and U-box-containing E3s, function as scaffolds for E2s and transfer ubiquitin directly to substrates; (3) RING1-between-RING2 (RBR) E3s, which function like RING/HECT hybrids, transfer ubiquitin-like HECT domain-containing E3s by binding to E2s via the RING1 domain and accept ubiquitin onto a cysteine in the RING2 domain (Fig. 1a).^12,32^

Ubiquitin itself can be ubiquitinated by other ubiquitin molecules at any one of its seven lysine residues (K6, K11, K27, K29, K33, K48, or K63) or the amino terminus (N-terminal methionine, M1) by forming isopeptide bonds between a lysine or methionine and the C-terminal glycine of adjacent ubiquitin moieties; therefore, homogeneous, heterogeneous or mixed chains are formed. Many kinds of chain linkages can be formed depending on which lysine (or methionine) is utilized for isopeptide bond formation: monoubiquitination; multiubiquitination; homotypic chains, including linear polyubiquitination (also known as Met1-linked chains),^33^ K6-, K11-, K27-, K29-, K33-, K48-, and K63-linked polyubiquitination; and heterotypic mixed or branched chains formed by mixed-linkage polyubiquitination.^10,11^ Different ubiquitin chains result in different biological outcomes. Specifically, monoubiquitination and multiubiquitination participate in protein-protein interactions and protein trafficking; K11- or K48-linked polyubiquitination, or K11-/K48-branched chains are related to proteasomal degradation; K63 linear polyubiquitination is associated with NF-κB and Akt signaling activation, protein complex formation, and endocytosis; K6 polyubiquitination is associated with the DNA damage response; K27 polyubiquitination contributes to mitochondrial maintenance and mitophagy; K29 polyubiquitination plays a role in lysosomal degradation; K33 polyubiquitination participates in TCR signaling; and Met1-linked linear ubiquitin chains are associated with NF-κB signaling and cell death.^31,32,34^ In addition, ubiquitin can be phosphorylated on its serine, threonine, and tyrosine residues or acetylated on its lysine residues. These ubiquitin modifications can fine-tune cellular signaling or participate in autophagy, mitophagy and other biological activities (Fig. 1b).^11,35,36^

The process of ubiquitination can be reversed via the removal of ubiquitin chains by a class of enzymes known as DUBs. This results in rescuing proteins from degradation or terminating ubiquitin signaling.^37^ More than 100 DUBs have been discovered in humans.^38^ DUBs can be divided into six major subclasses based on sequence homology, mechanism of action and structure of catalytic domains (Table 2).

**Table 2:** Classifications and functions of DUBs

Classification	Abbreviation	Full name	Functions	References
Cysteine proteases	USPs	Ubiquitin-specific proteases	(1) removal of ubiquitination to stabilize proteins and protect them from ubiquitination-proteasomal degradation; (2) processing and maturation of ubiquitin precursors; (3) cleaving poly-ubiquitin chains to recycle ubiquitin; (4) editing ubiquitin chains from one to another to adapt different activities.	^11,162,163^
	UCHs	Ubiquitin carboxyl-terminal hydrolases		
	OTUs	Otubain/ovarian tumor-domain containing proteins		
	MJDs	Machado-Joseph disease domain superfamily		
	MINDYs	Motif interacting with ubiquitin containing DUB family		
Zinc-dependent metalloproteases	JAMMs	JAB1/MPN/MOV34 proteases		

Discovering or developing drugs that target the process of ubiquitination or deubiquitination may herald the emergence of a new era of treating many challenging diseases since ubiquitination/deubiquitination is involved in the majority of physiological events. Disruption of the ubiquitination/deubiquitination pathway would definitely lead to detrimental outcomes.

## Targeting immune checkpoint pathways via the regulation of ubiquitination/deubiquitination

It has been widely reported that ubiquitination and deubiquitination are involved in many aspects of immune regulation, including TCR signaling, anergy, T cell differentiation, immune tolerance, and signal transduction.^31^ Increasing lines of evidence have indicated potential interactions between immune checkpoint pathways and ubiquitination/deubiquitination in cancers, infectious diseases, and autoimmune diseases. We will discuss these interactions and their clinical implications in the following part of the review.

### PD-1/PD-L1 pathway

#### The Cbl family

**Fig. 2:**
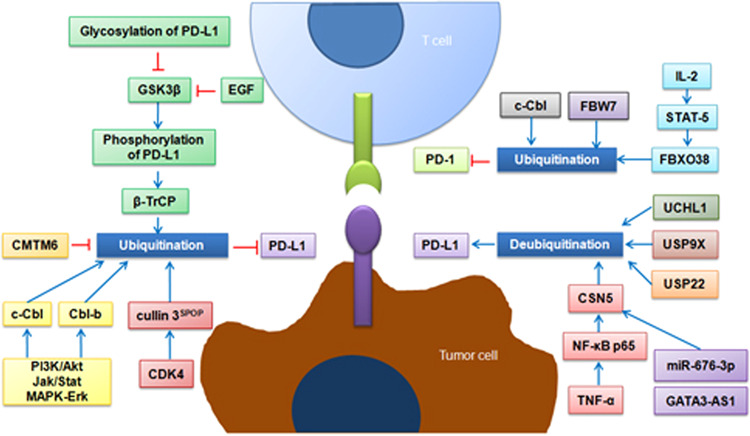
Schematic diagram of the regulation of PD-1/PD-L1 pathway by modulating ubiquitination/deubiquitination

The Casitas B-lineage lymphoma (Cbl) proteins are members of the RING-type E3 ligases, and there are three major isoforms of Cbl, namely, c-Cbl, Cbl-b, and Cbl-c.^39,40^ The Cbl proteins have a highly conserved N-terminal tyrosine kinase-binding (TKB) domain that binds to phosphorylated tyrosine residues, and a C3HC4 RING finger domain that is required for its E3 catalytic activity. C-Cbl and Cbl-b have relatively longer C-terminal proline-rich regions and a ubiquitin-associated (UBA) domain that bind to SH3 proteins and enable homodimerization, respectively. In contrast, Cbl-c has a shorter proline-rich region and lacks a UBA domain.^41^ The significance of the Cbl family in regulating the response to ICB treatment lies in the notion that they are capable of regulating PD-1/PD-L1 expression in cancers (Fig. 2). For instance, the deficiency of c-Cbl in colorectal cancer upregulates the expression of the PD-1 protein in tumor-infiltrating CD8 + T-lymphocytes and macrophages. The C-terminus of c-Cbl interacts with the cytoplasmic tail of PD-1, leading to PD-1 ubiquitination and proteolysis.^42^ Moreover, both c-Cbl and Cbl-b downregulate PD-L1 expression after the inhibition of PI3K/Akt, Jak/Stat, and MAPK-Erk signaling.^43^ These findings suggest that c-Cbl/Cbl-b is negatively associated with PD-1/PD-L1 expression and that targeting c-Cbl/Cbl-b may sensitize cancer patients to ICB treatments.

#### The SCF complex

The Skp1-cullin 1-F-box (SCF) RING-type E3 ligase complex is the largest family of E3 ligases and comprises of S-phase kinase-associated protein 1 (Skp1), ligase RING box 1 (Rbx1), cullin 1 (Cul1), and variable F-box proteins. Skp1 is a connexin that recruits the F-box protein to Cul1; Rbx1 contains the RING domain and binds to the E2-ubiquitin conjugate; Cul1 serves as a scaffold that connects Skp1 and Rbx1; F-box proteins are components that recognize protein substrates and determine the specificity of the SCF complex.^44–46^

Meng and colleagues discovered that a novel F-box protein that ubiquitinates the PD-1 protein. In their study, they found that activation of IL-2-STAT5 signaling activates the transcription of F-box only protein 38 (FBXO38). The function of this F-box protein has not been fully understood, however, the authors demonstrated that FBXO38 promotes the internalization, subsequent ubiquitination, and proteasomal degradation of the surface PD-1 protein in CD8+ T cells, in which the PD-1 protein undergoes Lys48-linked polyubiquitination at the Lys233 residue within the cytoplasmic domain (Fig. 2).^47^ Moreover, another F-box protein, F-box and WD repeat domain-containing 7 (FBW7), also regulates the ubiquitination and proteolysis of PD-1 protein (our unpublished data). This E3 ligase has been implicated in regulating the sensitivity to anti-tubulin chemotherapeutic agents through promoting MCL-1 protein ubiquitination and destruction.^48,49^ Interestingly, most FBW7’s substrates are oncogenic proteins associated with tumor progression and resistance to apoptosis. We reported that disruption of the FBW7-MCL-1 pathway leads to resistance to targeted therapy in NSCLC.^26^ We also identified transcriptional factor Snail as a novel substrate of FBW7. Loss of FBW7 expression results in increased Snail protein abundance, thereby causing epithelial-mesenchymal transition and tumor metastasis.^27^ More strikingly, a very recent study highlighted that inactivation of FBW7 was associated with altered immune microenvironment, decreased tumor-intrinsic expression of the double-stranded RNA (dsRNA) sensors melanoma differentiation-associated protein 5 (MDA5) and retinoic acid-inducible gene I (RIG-I), and diminished induction of type I IFN and MHC-I expression. Therapeutic reactivation of these pathways improves clinical responses to immunotherapy in FBW7 mutant in melanoma patients.^50^ Of noted, the substrate proteins need to be phosphorylated before being recognized by the SCF complex. The Ser159 and Thr163 residues of MCL-1 protein are primarily phosphorylated by glycogen synthase kinase 3β (GSK3β). The phosphorylated MCL-1 protein undergoes cytoplasm to nucleus translocation, where it binds to FBW7 E3 ligase.^26^

For the ubiquitination of PD-L1 protein, GSK3β-mediated phosphorylation also primes its interaction with β transducin repeat-containing protein (β-TrCP) E3 ubiquitin ligase, which is followed by proteasome-dependent degradation. However, only non-glycosylated PD-L1 interacts with GSK3β, and cell growth factors, including epidermal growth factor (EGF), could stabilize the PD-L1 protein through the suppression of GSK3β kinase activity.^51,52^

#### Other molecules/modifications associated with the (de)ubiquitination of PD-L1

In contrast to PD-1, the E3 ligases that degrade the PD-L1 protein largely remain to be determined. Interestingly, a deubiquitinase that stabilized the PD-L1 protein was identified prior to the E3 ligases that destroy the protein. A research team from UT MD Anderson Cancer Center reported COP9 signalosome 5 (CSN5) as a deubiquitinase for PD-L1 in 2016. In this study, the authors demonstrated that proinflammatory cytokines, such as tumor necrosis factor-α (TNF-α), stabilize the PD-L1 protein through NF-κB p65-CSN5 signaling activation (Fig. 2). CSN5 directly deubiquintinates the PD-L1 protein and the MPN domain of CSN5 is required for this process. Induction of CSN5 enables cancer cells to escape immune surveillance; thus, it is reasonable to believe that inhibition of CSN5 activity enhances the anticancer efficacy of immunotherapy.^53^ After the publication of this pioneering study, there was increasing interest in identifying the epigenetic mechanism of CSN5 regulation. For example, a very recent study showed a high level of GATA-binding protein 3 antisense RNA 1 (GATA3-AS1) in triple negative breast cancer, GATA3-AS1 sequesters miR-676-5p and increases the expression of CSN5.^54^ Moreover, ubiquitin-specific peptidase 9, X-linked (USP9X),^55^ ubiquitin-specific peptidase 22 (USP22)^56^ and ubiquitin C-terminal hydrolase L1 (UCHL1)^57^ have been demonstrated to deubiquitinate and stabilize the PD-L1 protein. Inactivation of these deubiquitinases readily elicits tumor suppressive effects in various cancer cell lines. There are also adaptor proteins (not deubiquitinases) that maintain PD-L1 protein abundance. Two independent research groups identified CKLF-like MARVEL transmembrane domain-containing protein 6 (CMTM6) as a critical regulator of PD-L1 protein expression. The authors showed that CMTM6 co-localizes with the PD-L1 protein at the plasma membrane and in recycling endosomes, where it prevents the PD-L1 protein from being ubiquitinated. CMTM6 enables PD-L1-expressing cancer cells to escape T cell-mediated anti-tumor immunity, whereas CMTM6 depletion significantly reduces the protein expression of PD-L1 and alleviates the suppression of tumor-specific T-cell activity.^58,59^ Thus, targeting these deubiquitinases or protein adaptors is believed to enhance the efficacy of immunotherapy by facilitating PD-L1 protein degradation.

Several cytokines and protein kinases have been shown to antagonize deubiquitinases and induce PD-L1 protein ubiquitination. Previous studies have confirmed that EGF treatment activates PD-L1 mRNA transcription, while EGF also triggers PD-L1 posttranslational modification. Horita and colleagues reported that EGF induces the mono- and multiubiquitination of PD-L1 and precedes EGF-induced increases in the PD-L1 mRNA level.^60^ This finding partially explains the low expression of the PD-L1 protein in EGFR mutant cancer cells, although the activation of EGFR signaling leads to the transcription of PD-L1 mRNA. The exact E3 ligase that ubiquinates the PD-L1 protein was discovered by Wei’s lab in 2017.^61^ In this study, they provided direct evidence showing that Cullin 3^SPOP^ (speckle-type POZ protein) is the physiological E3 ligase for the PD-L1 protein, by which the C-terminal tail of PD-L1 protein (283–290) binds to the substrate-interacting MATH domain of SPOP (Fig. 2). Cancers carrying mutant SPOP displayed elevated PD-L1 levels and significantly reduced CD3+ TIL numbers in the tumor microenvironment. Moreover, the authors highlighted that SPOP and PD-L1 converge at cyclin-dependent kinase 4/6 (CDK4/6). Inhibition of CDK4/6 by commercially available small molecule inhibitors readily suppresses the E3 ligase activity of SPOP, and consequently stabilized the PD-L1 protein. These findings may provide a complementary molecular rationale for combining CDK4/6 inhibitors with immunotherapy as a novel strategy because the efficacy of PD-1/PD-L1 blockade correlates with the expression levels of PD-L1 in tumor cells. Taken together, deciphering the regulatory machinery upstream (e.g., transcription) and downstream (e.g., ubiquitination/deubiquitination) of PD-1/PD-L1 would definitely provide novel therapeutic targets to enhance the efficacy of immunotherapy. The combination with small molecule inhibitors that directly target either ubiquitinases/deubiquitinases or PD-1/PD-L1 protein adaptors would represent a state-of-art approach for cancer patients.

### CTLA4/B7 pathway

CD80 (B7-1) and CD86 (B7-2) are both type 1 transmembrane proteins that contain a membrane distal IgV and a membrane proximal IgC domain. These proteins belong to the B7 immunoglobulin superfamily and are primarily expressed on APCs. These proteins bind to CD28 to activate costimulatory signaling and enable the full activation of T cells, whereas the binding of CTLA-4 to CD28 antagonizes B7-CD28 signaling, thus, suppresses T cell activation and maintains the homeostasis of immune system.^62,63^

The exact E3 ubiquitin ligases that degrade CTLA-4 protein have not been identified at the moment. However, there are preliminary evidence showing potential casual relationships between CTLA4 protein abundance and E3 ubiquitin ligases.

**Fig. 3:**
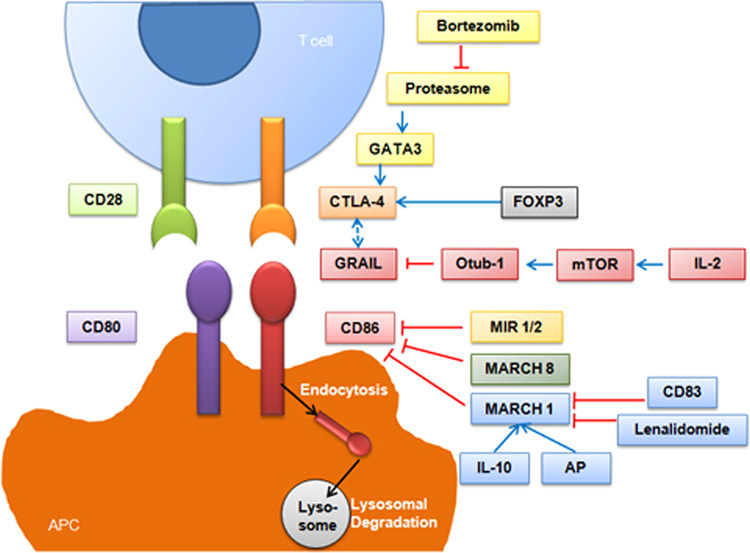
Schematic diagram of the regulation of CTLA4/B7 pathway by modulating ubiquitination/deubiquitination

In the *Trypanosoma cruzi* (*T. cruzi*) infection-induced CD4+ T cell unresponsiveness model, infection with *T. cruzi* led to a significant increase in the expression of both CTLA-4 and PD-1 in spleen CD4+ T cells, which might be attributed to the upregulation of the E3 ubiquitin ligase GRAIL (gene related to anergy in lymphocytes) during infection. Indeed, the T cell anergy during infections is characterized by a lack of cytokine responses and reduced proliferative activities, which can be reversed by the addition of IL-2. IL-2 treatment activates the mammalian target of rapamycin (mTOR) pathway and induces Otubain-1 expression, which mediates GRAIL degradation and improves T cell proliferation.^64,65^ Furthermore, the dysfunction of proteasome would lead to accumulation of GATA-binding protein 3 (GATA3), which transcriptionally activates CTLA-4 and inhibits T-cell responses in T cell malignancy (Fig. 3).^66^

A large number of studies have demonstrated that modulators of immune recognition MIR1 (K3) and MIR2 (K5) encoded by Kaposi’s sarcoma-associated herpesvirus (KSHV) are members of viral membrane-associated RING-CH (MARCH) E3 ubiquitin ligases.^67^ MARCH E3 ligases contain a zinc-finger domain and plant homeobox domain (PHD), which mediate the ubiquitination of cell-surface proteins like MHC-I, B7-2, and intercellular adhesion molecule-1 (ICAM-1). Degradation of these membrane proteins results in impaired recognition by host cytotoxic T lymphocytes, and leads to immune evasion.^68–73^ Interestingly, viral MARCH E3 ligases not only ubiquinate on the lysine residues of substrates, but also on the cysteine, serine or threonine residues.^74–76^

The MARCH family, which is the cellular orthologs of MIR1 and MIR2, all contains the C4HC3 configuration of cysteines and histidine; this family includes 11 members that function similarly to MIR1 and MIR2.^77^ Cellular MIR (c-MIR), also termed as MARCH VIII,^78^ participates in the ubiquitination, endocytosis, and lysosomal degradation of B7-2 (Fig. 3).^79^

Other MARCH family members, such as MARCH 1, participate in regulating the immune response and could be manipulated by pharmacological approach as well. For example, Foxp3+ Tregs elicit the immunosuppressive effect on DCs through the binding of CTLA-4 expressed on Tregs and CD80/CD86 on DCs.

While CD86 and MHC-II expression could be ubiquitinated by MARCH 1.^80–82^ This posttranslational modification could be readily enhanced by IL-10^83–85^ and apple polyphenol extract (AP) treatment,^86^ or suppressed by CD83^83,87^ and lenalidomide (Fig. 3).^88^ MARCH 1 has also been documented to be regulated by itself through dimerization and autoubiquitination.^89^ However, another study reported contradictory results, that the ubiquitination and degradation of MARCH 1 are mediated by an unknown E3 ligase with the help of ubiquitin-conjugating enzyme E2 D1 (Ube2D1), rather than MARCH 1 itself.^90^

### Other immune checkpoint pathways modulated by ubiquitination/deubiquitination

#### LAG-3 and its ligands

As mentioned above, LAG-3 is also an immune checkpoint that predominantly interacts with MHC-II, fibrinogen-like 1 (FGL1), galectin-3, C-type lectin-like domain family 4, member g (LSECtin), and α-synuclein.

**Fig. 4:**
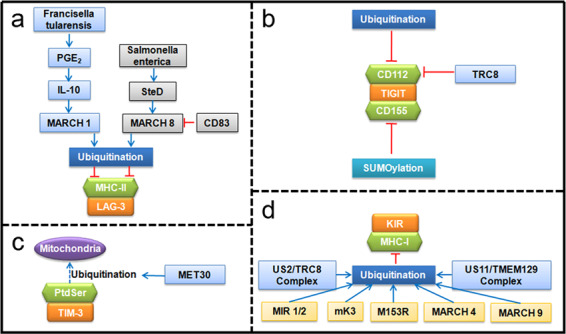
Schematic diagram of the regulation of **a** LAG-3, **b** TIGIT, **c** TIM-3, and **d** KIR checkpoint pathways by modulating ubiquitination/deubiquitination

Proteins of the MARCH family, including MARCH 1 and MARCH 8, have been reported to downregulate the cell-surface expression of MHC-II in DCs,^91–94^ B cells,^95,96^ and monocytes^97^ by ubiquitinating lysine residues in the cytoplasmic tail of MHC-II β-chains.^84,98,99^ These findings frequently occurred during infection. The intracellular bacterium *Francisella tularensis* (*F. tularensis*) survives and replicates within macrophages, which induces prostaglandin E2 (PGE2) production and immune tolerance through MARCH E3 ligase-mediated ubiquitination of MHC-II.^100,101^ Similarly, *Salmonella enterica* (*S. enterica*) evades immune surveillance by the SteD and MARCH 8-dependent ubiquitination of MHC-II (Fig. 4a).^102^

In contrast, compensatory mechanisms maintain the expression of MHC-II. Scientists have identified CD83 as necessary and sufficient for thymic CD4 T cell selection, during which CD83 antagonizes MARCH 8 E3 ligase to stabilize MHC-II; genetic ablation of MARCH 8 in Cd83^−/−^ mice restored CD4 T cell development.^103,104^

#### TIGIT and its ligands

CD112 (also called nectin-2, PRR2, or PVRL2) is an adhesion molecule of the immunoglobulin superfamily. CD155 (also called PVR or Necl-5) is a member of the nectin-like molecule family and functions as an adhesion molecule. Both CD112 and CD155 are upregulated in virus-infected cells or in tumor cells and modulate the activation or inhibition of NK cell-mediated cytolysis by binding to CD226 or TIGIT.^105,106^

Molfetta and colleagues reported that CD112 undergoes proteolysis through ubiquitination and that inhibition of the ubiquitin pathway increases its cell-surface expression, which enhances the efficacy of NK cells in killing tumor cells.^106^ TRC8 is the RING E3 ligase that recognizes CD112 as a substrate.^107^

Unlike CD112, CD155 undergoes SUMOylation, which adds the small ubiquitin-like modifier (SUMO) protein, rather than ubiquitin, to lysine residues of substrates (Fig. 4b). Interestingly, inhibition of SUMOylation promotes the translocation of CD115 from the cytoplasm to the cell membrane.^108^ This has potential clinical implications for treating CD115-expressing cancers because the translocation of CD115 to the cell surface clearly facilitates tumor cell recognition by innate immune cells and increases tumor cell susceptibility to NK cell-mediated cytolysis.

#### TIM-3 and its ligands

Phosphatidylserine (PtdSer) is one of the ligands of TIM-3 and is a phospholipid that is abundant in eukaryotic plasma membranes and participates in apoptosis and blood clotting.^109^ A study has reported that the transport of PtdSer from the endoplasmic reticulum to mitochondria is regulated by ubiquitination; this phenomenon is mediated by the MET30 gene, which encodes a substrate recognition subunit of SCF ubiquitin ligase (Fig. 4c).^110^

#### KIR and MHC-I

Killer cell immunoglobulin-like receptors (KIRs) are a set of polymorphic transmembrane glycoproteins that are mainly expressed on NK cells; KIRs serve as both activating and inhibitory receptors and play crucial roles in the regulation of NK cell functions.^111^ MHC-I is currently the only identified ligand of KIR. In agreement with the process of ubiquitination of MHC-II and CD86, MIR 1, MIR 2, murine γ-herpesvirus-68 K3 (mK3), M153R, MARCH 4 and MARCH 9 have been demonstrated to downregulate the expression of MHC-I mainly through Lysine-63-linked ubiquitination on the cytoplasmic tail of the MHC-I protein, followed by its internalization, endocytosis, and lysosomal degradation.^112–121^ Viral MARCH E3 ligases can also ubiquitinate MHC-I without the presence of cytosolic tail lysines.^120^ The TRC8/US2 complex also leads to the polyubiquitination and proteasomal degradation of MHC-I.^167^ Another ER-resident membrane protein, TMEM129, which is nonclassical RING E3 ligase, binds to US11 and mediates the ubiquitination of MHC-I (Fig. 4d).^107,122^

#### B7-H4

B7-H4 is a type I transmembrane protein and a member of the coinhibitory B7 family ligands. B7-H4 has been found to play an inhibitory role in immune responses and contribute to poor prognosis of multiple tumors.^123,124^ Recent research has reported that NGI-1 inhibits the glycosylation of B7-H4, leading to the ubiquitination of B7-H4 by the E3 ligase autocrine motility factor receptor (AMFR). The removal of B7-H4 would enhance anti-tumor immunity and promote immunogenic cell death. In addition, the authors verified the effectiveness of a triple combination of NGI-1, camsirubicin (a chemotherapeutic agent that increases the immunogenicity of tumor) and PD-L1 blockade in treating tumors in preclinical breast cancer models.^125^ To this end, targeting the immunosuppressive molecule B7-H4 would be a novel strategy for facilitating anticancer immunity.

### Drugs that regulate immune checkpoint pathways by modulating (de)ubiquitination

The drugs mentioned in this review that may help to regulate immune checkpoint pathways by regulating the process of ubiquitination or deubiquitination are listed in the table below (Table 3 and Table 4).

**Table 3:** Drugs that may regulate the immune checkpoint pathways through targeting ubiquitination/deubiquitination (clinical)

Type	Name	Target	Developer	Implications	Stage
Proteasome inhibitors	Bortezomib (Velcade, PS-341, MLN-341)	20S core subunit	Millennium	Relapsed and/or refractory multiple myeloma; mast cell leukemia	Approved
	Carfilzomib (Kyprolis, PR-171)	20S core subunit	ONYX	Relapsed and/or refractory multiple myeloma	Approved
	Ixazomib (Ninlaro, MLN-9708)	20S core subunit	Millennium	Relapsed and/or refractory multiple myeloma; acute myeloid leukemia; follicular lymphoma; peripheral T-cell lymphoma	Approved
	Marizomib (NPI-0052, Salinosporamide A)	20S core subunit	Nereus	Relapsed and/or refractory multiple myeloma; non-small cell lung cancer; pancreatic cancer; melanoma; lymphoma; ependymoma; glioblastoma	Phase 3
	Oprozomib (ONX-0912, PR-047)	20S core subunit	ONYX	Multiple myeloma; Waldenstrom Macroglobulinemia; solid tumors; advanced non-central nervous system malignancies	Phase 1/2
	Delanzomib (CEP-18770)	20S core subunit	Cephalon	multiple myeloma; solid tumors; non-Hodgkin lymphoma	Phase 1/2
E1 inhibitors	MLN-7243 (TAK-243)	Ubiquitin E1 enzyme	Millennium	myelodysplastic syndrome; acute myeloid leukemia; myelomonocytic leukemia; advanced malignant solid tumors	Phase 1
	Pevonedistat (MLN-4924, TAK-924)	NEDD8 activating enzyme	Millennium	Acute myeloid leukemia; myelodysplastic syndrome; plasma cell myeloma; metastatic melanoma; solid tumors	Phase 3
E3 inhibitors	Lenalidomide (Revlimid)	MARCH 1	Celgene	Multiple myeloma; myelodysplastic syndromes; mantle cell lymphoma	Approved

**Table 4:** Drugs that may regulate the immune checkpoint pathways through targeting ubiquitination/deubiquitination (preclinical)

Type	Name	Target	Preclinical models	References
Proteasome inhibitors	MG132	20S core subunit	In vitro	^61,127^
	Epoxomicin	20S core subunit	In vitro	^128^
	Lactacystin	20S core subunit	In vitro	^128^
E1inhibitors	PYR-41	ubiquitin E1 enzyme	In vitro	^60^
E3 agonists	Apple polyphenols	MARCH 1	THP-1-derived human DCs	^86^
DUB inhibitors	Curcumin	CSN5	Breast cancer melanoma colon cancer	^53^
Others	CDK4/6 inhibitors	Cullin 3^SPOP^	Prostate cancer	^61^
	STAT/AKT/ERK inhibitors	c-Cbl, Cbl-b	Lung cancer	^43^
	EGFR inhibitors	GSK3β, β-TrCP	Breast cancer	^52^

The ubiquitin proteasome system (UPS) is the common destination for the degradation of ubiquitinated proteins. The 26S proteasome is composed of one 20S core subunit and two 19S regulatory subunits. The 20S core subunit plays a major role in protein degradation and includes three catalytic sites that exhibit chymotrypsin-like (β5), trypsin-like (β2), and caspase-like (β1) activities. The 19S subunits modulate the deubiquitination of proteins. Therefore, proteasome inhibitors mainly target the 20S core subunit to impair its proteolytic activity.^37,126^

Recent decades have witnessed the successful application of many proteasome inhibitors to clinical practice, mainly in hematologic malignancies. In addition, MG132, epoxomicin, and lactacystin, which are widely used in preclinical studies, have been found to be effective in regulating the stability of immune checkpoints in vitro.^61,127,128^ However, treatment with proteasome inhibitors may lead to broad-spectrum adverse effects, such as peripheral neuropathy, nausea, vomiting, and heart failure. In addition, the impairment of the UPS may result in the accumulation of redundant “rubbish” proteins.^126^ These concerns contribute to the limited clinical applications of proteasome inhibitors.

E1 inhibitors MLN-7243 and MLN-4924 have also been tested in the clinic. Unfortunately, these drugs target the first step of ubiquitination and cause numerous adverse effects.^129^ On the other hand, targeting E2 conjugating enzymes by small molecule inhibitors, including CC0651,^130^ NSC697923^131^ and BAY 11–7082,^132^ is expected to be clinically beneficial. However, the results are rather disappointing, as none of these drugs have entered clinical trials or have been reported to have an effect on the regulation of immune checkpoint pathways. The failure of targeting E1 and E2 suggests that the upstream inhibition of protein ubiquitination may not be practical, however, downstream inhibition or activation of protein ubiquitination, such as by targeting E3 ligases or DUBs, may be alternative options due to their high substrate specificity. Indeed, this strategy is effective in both preclinical and clinical settings. For example, the E3 agonist BM that targets SLIM has shown therapeutic efficacy in a mouse model of autoimmune encephalomyelitis;^133^ oridonin, which targets FBW7, induces cancer cell apoptosis and overcomes resistance to targeted therapy.^26^ The E3 inhibitor lenalidomide, which targets MARCH 1, has been used to treat hematologic malignancies;^88^ the DUB inhibitor curcumin destabilizes CSN5 and improves the efficacy of immunotherapy in breast cancer, melanoma, and colon cancer;^53^ P5091, which targets USP7, induces apoptosis in multiple myeloma cell lines.^134^ Given the success of these E3 inhibitors and DUB inhibitors, increasing efforts have been made to design and develop novel small molecule inhibitors that are expected to modulate immune checkpoint pathways. Further randomized clinical trials are warranted to evaluate the safety and efficacy of these novel inhibitors in cancer patients who receive immunotherapy.

To promote the efficiency of the degradation of proteins of interest (POIs) related to immune checkpoint pathways, especially the degradation of undruggable proteins, a variety of new techniques can be applied and proteolysis targeting chimeras (PROTACs) are useful tools that were developed in recent years. PROTACs were first reported by Kathleen M. Sakamoto and act as a bridge between E3 ligases and targeted proteins to enhance the degradation of substrate proteins through the UPS.^135^ However, PROTACs still face many challenges. The primary problem is the poor oral bioavailability of PROTACs due to their large and complex structure. Furthermore, PROTACs may degrade target proteins in both normal and tumor cells, which will cause a series of side effects. In addition, the hook effect of PROTACs should not be ignored. Moreover, it is difficult for PROTACs to target extracellular and membrane-associated proteins. To resolve these problems, advanced PHOTAC^136^ and opto-PROTAC^137^ have recently been developed. The two reports integrated photoswitches or ultraviolet A irradiation with PROTACs to achieve effective protein degradation. In addition, lysosome-targeting chimaeras (LYTACs) have been recently established to degrade extracellular and membrane-associated proteins by binding to a cell-surface lysosome-shuttling receptor and the extracellular domain of a protein. Scientists have demonstrated that LYTACs can degrade apolipoprotein E4, EGFR, CD71, and PD-L1.^138^ In summary, PROTACs and LYTACs have strong potential to exert positive effects on precision therapy that aims to degrade specifically proteins relevant to immune checkpoint pathways.

## Conclusions

### Conclusion and perspectives

As described throughout this review, numerous studies have provided important insights into the interactions between immune checkpoint pathways and ubiquitination or deubiquitination, allowing us to understand the great potential of targeting the ubiquitin system and immune checkpoints along with using as novel therapeutic strategies. Since the therapeutic effects and response rates of immunotherapy are currently relatively low, this therapy may provide more options for those who cannot benefit from the current therapies. In addition, with the development of sequencing techniques and bioinformatics, an increasing number of genes associated with (de)ubiquitination that exhibit different expression patterns in tumor tissues and normal tissues will be identified to help us design specific and safe interventions. Although the vast majority of the drugs mentioned above have only been studied in animal models or in vitro and the development of relevant drugs and their translation to clinical applications still requires substantial efforts, there is abundant evidence that targeting the ubiquitination/deubiquitination process to regulate immune checkpoint pathways has great potential for providing more therapeutic options for those who suffer from cancers, infections, and autoimmune diseases in the future.
